# Prospective study of the effect of auricular percutaneous electrical nerve field stimulation on quality of life in children with pain related disorders of gut-brain interaction

**DOI:** 10.3389/fpain.2023.1223932

**Published:** 2023-09-08

**Authors:** Ashish Chogle, Kaajal Visnagra, Jamie Janchoi, Tammy Tran, Rachel Davis, Nicole Callas, Elisa Ornelas

**Affiliations:** ^1^ Division of Pediatric Gastroenterology, Hepatology and Nutrition, CHOC Children’s, Orange, CA, United States; ^2^ Department of Pediatrics, University of California Irvine, Orange, CA, United States; ^3^ Research Institute, CHOC Children’s, Orange, CA, United States

**Keywords:** irritable bowel syndrome, percutaneous electrical nerve field stimulation, gastrointestinal disorder, auricular, quality of life, disorder of gut brain interaction, functional abdominal pain disorder

## Abstract

**Background:**

Disorders of the Gut-Brain Interaction (DGBIs) account for 50% of pediatric gastrointestinal (GI) consultations. Children with DGBIs have worse quality of life (QoL) than those with organic GI disorders such as inflammatory bowel disease and gastroesophageal reflux disease. Pediatric DGBIs patients, especially those with chronic abdominal pain (AP), have impaired QoL and increased psychological distress in the form of anxiety and depression. Percutaneous Electrical Nerve Field Stimulation (PENFS) therapy has been shown to be effective in improving symptoms and functioning in children with DGBIs. The treatment's impact on these patients' QoL is unknown.

**Methods:**

This prospective study evaluated changes in QoL, gastrointestinal symptoms, functional disability, somatization, global health, anxiety, and depression in patients aged 11–18 years who received PENFS therapy (IB-stim, NeurAxis, Versailles, IN) for treatment of pain related DGBIs, once a week for four consecutive weeks.

**Results:**

This study included 31 patients with an average age of 15.7 years (SD = 2); 80.6% were female. After PENFS therapy, patients reported significant reductions in abdominal pain, nausea severity, functional disability, somatization, and anxiety from baseline to week 4 (*p* < 0.05). Parents reported significant improvement in their child's QoL regarding physical function, psychosocial function, and generic core scale scores (*p* < 0.05). Parents also noted reduced abdominal pain, functional disability, and somatization. Average scores on the Patient-Reported Outcomes Measurement Information System (PROMIS) Global Health scale significantly improved based on both patient and parent reports (*p* < 0.05). Our patients' QoL was significantly lower than healthy controls at baseline and after treatment (*p* < 0.05).

**Conclusion:**

Our research demonstrates that PENFS significantly enhances the QoL of children suffering from pain-related DGBIs, in addition to improvement in GI symptoms, daily functioning, somatization, global health, and psychological comorbidities. These findings demonstrate the effectiveness of PENFS and its potential to alleviate the suffering of countless children.

## 1. Introduction

DGBIs is prevalent in as many as 25% of children around the globe (1). These patients account for up to 50% of all pediatric gastroenterology clinic visits in the US (2). Children with DGBIs report significantly lower quality of life (QoL) scores than healthy peers and children with organic GI disorders (3). DGBIs are ranked among the leading causes of school absences (4). A large proportion of children with DGBIs continue to have symptoms into adulthood (5). Pediatric DGBIs patients have impaired QoL, psychological distress in the form of anxiety and depression, and functional disability (3). There is a huge healthcare burden associated with these conditions (6). It is a daunting task to treat pediatric DGBIs as there is a lack of effective therapies currently available. The initial course of action usually involves nonpharmacologic treatments and lifestyle interventions (7). If the symptoms do not improve, doctors may prescribe antidepressants. However, it's important to note that these medications are not FDA-approved for treating DGBIs and may have severe side effects (8). Currently, there is inadequate proof to support the use of medications for children experiencing DGBIs.

There is increasing evidence about the efficacy of auricular percutaneous electrical nerve field stimulation (PENFS) for the treatment of pediatric DGBIs. PENFS is the only FDA-approved treatment for this indication (9). PENFS acts via non-invasive electrical stimulation of the auricular branches of the vagus nerve and eventual modulation of central pain pathways (10). An improvement in abdominal pain, functioning, and overall well-being was seen after 3 weeks of treatment with PENFS in a large randomized controlled trial in adolescents with DGBIs. Sustained improvement and minimal side effects were reported (11, 12). An open-label study in adolescent DGBIs reported improved pain, nausea, sleep, disability, and anxiety after four weeks of PENFS therapy with some effects sustained for 6–12 months (13).

## 2. Material and methods

**Figure 1:**
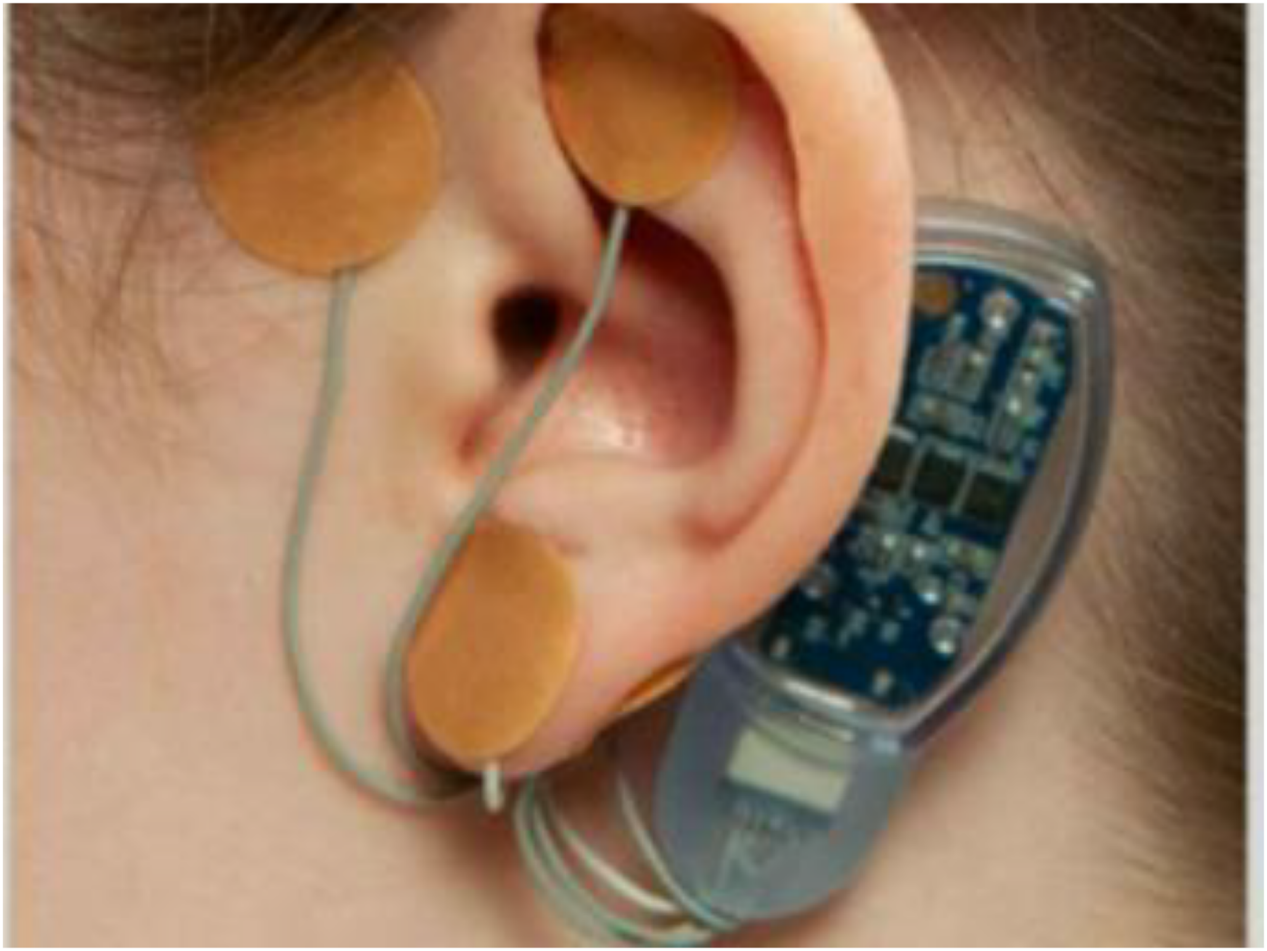
Image of PENFS.

This prospective observational study evaluated QoL in children aged 11–18 years at CHOC Children's who received PENFS therapy (IB-stim, NeurAxis, Versailles, IN), from September 2020 to September 2022, for treatment of pain related DGBIs, once a week for four consecutive weeks. All study patients were fluent in English and had access to a cell phone/tablet/laptop or computer, with at-home access to an internet connection. Consent was obtained from all study participants prior to participation in the study. The study was approved by the institution's review board (IRB# 200343). Figure 1 demonstrates shows an image of PENFS device.

Demographic information including age and gender was recorded at baseline, along with the results of the clinical evaluation. The QoL questionnaire was completed by parent-child dyads at baseline and at Week 4. Patients completed the PedsQL Generic Core Scales and the PedsQL General well-being scale and both are scored on an average scale from 0 to 100 (ages 8–12 or 13–18 years). Parents completed the parent-proxy report of the PedsQL Generic Core Scales and the PedsQL General well-being scale (ages 8–12 or 13–18 year).

Children reported symptoms using the Abdominal Pain Index (API) scored on an average scale from 0 to 5, Nausea Severity Scale (NSS) scored on an average scale from 0 to 4.25, and the Functional Disability Inventory (FDI) scored on an sum form 0 to 60, which is a one-page measure of the difficulty experienced by the child in physical and psychosocial functioning due to impaired physical health. The Child Somatization Inventory (CSI) scored on a sum from 0 to 96 and was used to evaluate somatization, which is the physical expression of stress and emotions. At baseline and upon completion of PENFS therapy, patients were evaluated using Patient-Reported Outcomes Measurement Information System (PROMIS) Global Health Anxiety, and Depression scored on a sum from 7 to 35, while PROMIS Anxiety and PROMIS Depression scored on a sum from 8 to 40; parent-reported answers were obtained as well. All data collected was entered by a member of the research team in the password-protected REDCap database. A dataset on QoL in healthy pediatric controls reported by Varni JW et al. was used for comparison (3).

For the questionnaires used, the lower score indicates better health outcomes and higher score indicate worse health outcomes. The exception is the QoL questionnaire where higher score indicate better QoL. A timeline of questionnaire completion is demonstrated in Table 1.

**Table 1:** Timeline of events.

Questionnaires	Baseline questionnaires	Weekly PENFS therapy
Demographics	x	
Medical history	x	
Rome IV diagnostic questionnaire	x	
Quality of life generic core scale and general well-being scale	x	x
Abdominal pain index (API)	x	x
Nausea severity scale (NSS)	x	x
Child somatization inventory (CSI)	x	x
Functional disability inventory (FDI)	x	x
Patient-reported outcomes measurement information system (PROMIS) (Depression, anxiety and global health)	x	x

### 2.1. Statistical analysis

*P*-value–based significance was calculated using a generalized mixed effects model (GLMM) with compound symmetry specified for repeat measures and variance components for random effect of patients. Normal distribution was assumed for all outcomes, with exception of FDI and NSS scores, that were positively skewed and had gamma distribution with log link utilized. Comparison of mean value in study cohort at each time point was compared to healthy controls based on Welch's *t*-test of two independent samples with unequal variance. Spearman's correlation coefficient was used to evaluate the correlation in symptoms and QoL and PROMIS outcomes.

## 3. Results

This study included 31 patients, ages from 11 to 18 years (mean 15.7 years, SD 2 years); 80.6% were female. The caregiver who provided data identified as the patient's mother in 77.4% of cases. Medication use was reported by 83.9% of patients, with median number of medications 5 (IQR: 1.0, 7.0) (Table 2). Out of the 31 patients, 17 (54%) reported minor side effects from device placements. These included itchiness (50%), soreness (27%), and pain at device site (22%). No severe side effects were reported. Disorders of gut-brain interaction (DGBIs) obtained, with 13 patients (41%) having Irritable Bowel Syndrome (IBS) and 9 (29%) patients with Functional Dyspepsia (FD).

**Table 2:** Characteristics of study population.

N (%), mean (SD), or median [IQR]	Overall
	*N* = 31
Patient age, years, mean (SD)	15.7 (2.0)
Patient gender	
Female	25 (80.6%)
Male	5 (16.1%)
Not specified	1 (3.2%)
Type(s) of medications	
NSAIDs	20 (64.5%)
Neuromodulators	5 (16.1%)
Anti-anxiety/antidepressants	7 (22.6%)
Antispasmodic	11 (35.5%)
Laxatives	5 (16.1%)
Opioids	2 (6.5%)
Antinausea	10 (32.3%)
Disorder of gut-brain interaction	
Irritable bowel syndrome	13 (41.9%)
Functional dyspepsia	9 (29.0%)
Number of medications, median [IQR]	5.0 [1.0, 7.0]
None	5 (16.1%)
One	3 (9.7%)
Two	2 (6.5%)
Three or more	20 (64.5%)
Not specified	1 (3.2%)
Caregiver filling out forms	
Mom	24 (77.4%)
Dad	1 (3.2%)
Other	3 (9.7%)
Not specified	3 (9.7%)

Patients reported significant average reductions in abdominal pain, nausea severity, functional disability, somatization, and anxiety from baseline to Week 4 (*p* < 0.05). Self-reported QoL and depression did not significantly change from baseline to Week 4 (*p* > 0.05). However, parents reported significant improvement in average QoL for their child in terms of physical function, psychosocial function, and generic core scale scores (*p* < 0.05). Parents also noted reduced average functional disability, abdominal pain, and somatization. Average scores on the PROMIS Global Health scale significantly improved based on both patient and parent report (*p* < 0.05). Adjustment for age, gender, and number of medications did not alter the direction nor significance of findings reported in the unadjusted analyses (Table 3).

**Table 3:** Average change in quality of life (QOL), nausea, FDI, child somatization inventory, API, and PROMIS metrics from baseline to 4 weeks based on child and parent responses, adjusted for patient age, gender, and number of medications (0, 1–2, 3+).

Outcomes	Baseline	Week 4	Difference (Wk4-Base) Mean (95% CI)	*P*-val^a^
	Mean (95% CI)	Mean (95% CI)		
Child reported:				
QOL				
Physical functioning	46.4 (38.1, 54.7)	46.5 (37.7, 55.3)	0.1 (−7.3, 7.6)	.973
Psychosocial	54.3 (46.6, 62.1)	57.7 (49.5, 65.9)	3.4 (−3.7, 10.5)	.346
Generic core scale	52.2 (44.9, 59.4)	53.6 (46.0, 61.2)	1.5 (−4.3, 7.2)	.612
General well being	64.2 (57.6, 70.8)	61.3 (54.3, 68.3)	−2.9 (−9.0, 3.3)	.350
Nausea severity	1.7 (1.3, 2.3)	1.1 (0.7, 1.5)	−0.6 (−1.1, −0.2)	.003*
Functional disability inventory	15.8 (11.5, 21.7)	11.8 (8.6, 16.2)	−4.0 (−7.0, −1.1)	.004*
Child somatization inventory	42.8 (36.0, 49.6)	25.6 (18.7, 32.4)	−17.3 (−22.0, −12.5)	<.001*
Abdominal pain index	3.4 (2.9, 3.9)	2.1 (1.6, 2.6)	−1.3 (−1.8, −0.8)	<.001*
PROMIS global health	20.0 (18.4, 21.6)	20.9 (19.3, 22.5)	0.9 (0.0, 1.8)	.054
PROMIS child anxiety	18.6 (15.7, 21.5)	16.2 (13.3, 19.1)	−2.4 (−4.2, −0.6)	.011*
PROMIS child depression	17.4 (14.4, 20.5)	15.3 (12.3, 18.4)	−2.1 (−5.1, 0.9)	.168
Parent reported:				
QOL				
Physical functioning	46.1 (37.1, 55.1)	56.0 (46.2, 65.9)	9.9 (0.7, 19.1)	.036*
Psychosocial	48.0 (41.3, 54.6)	60.0 (52.6, 67.5)	12.1 (3.9, 20.3)	.005*
Generic core scale	47.7 (40.8, 54.6)	58.8 (51.1, 66.4)	11.1 (3.6, 18.6)	.005*
General well being	56.2 (49.9, 62.5)	57.1 (50.2, 64.1)	0.9 (−5.4, 7.3)	.767
Nausea severity	1.3 (0.7, 2.0)	1.1 (0.6, 1.8)	−0.1 (−0.7, 0.4)	.575
Functional disability inventory	16.0 (11.1, 23.0)	11.8 (8.2, 17.0)	−4.2 (−8.3, −0.1)	.033*
Child somatization inventory	37.0 (28.8, 45.2)	25.9 (17.5, 34.3)	−11.1 (−17.5, −4.7)	.001*
Abdominal pain index	3.4 (2.8, 4.0)	2.2 (1.6, 2.8)	−1.2 (−1.7, −0.6)	<.001*
PROMIS global health	19.2 (17.2, 21.1)	20.9 (19.0, 22.8)	1.7 (0.7, 2.8)	.002*

* *p* < .05.

^a^ *P*-value based significance on generalized mixed effects model (GLMM) with compound symmetry specified for repeat measures and variance components for random effect of patients. Normal distribution assumed for all outcomes with exception of FDI and Nausea which were positively skewed and gamma distribution with log link utilized (nausea scale included zero values, therefore, one was added to scale score and back-transformed for above presentation of estimates from model).

QOL scales: higher scores suggest better quality of life.

The number of medications patients were taking influenced several outcomes. Anxiety decreased significantly from baseline to Week 4 in patients on greater than or equal to 3 medications (*p* < 0.05); no significant change in anxiety was observed in patients not on medication. Similarly, nausea severity decreased, on average, in those on greater than or equal to 3 medications, with minimal change observed in those on fewer medications. QoL physical functioning according to parent report also improved, on average, in patients on greater than or equal to 3 medications, with no significant differences in those on fewer medication (Table 4).

**Table 4:** Description of factor level dependency in average change for respective outcomes from baseline to 4 weeks, adjusted for patient age, gender, and number of medications (0, 1–2, 3+) (interaction effect, *p* < .10).

Outcomes	Baseline	Week 4	Difference (Wk4-Base) Mean (95% CI)	*P*-val^a^
	Mean (95% CI)	Mean (95% CI)		
Child reported				
QOL general well being				
0 medications	50.1 (33.7, 66.4)	60.4 (43.4, 77.4)	10.3 (−1.3, 21.9)	.079
1–2 medications	63.1 (43.9, 82.4)	70.6 (52.3, 88.9)	7.5 (−5.9, 20.8)	.266
3 + medications	67.4 (59.2, 75.5)	58.7 (50.1, 67.3)	−8.7 (−14.6, −2.7)	.005*
PROMIS child anxiety				
0 medications	18.5 (11.7, 25.3)	20.8 (13.8, 27.8)	2.3 (−1.8, 6.4)	.269
1–2 medications	15.7 (9.0, 22.5)	12.1 (5.4, 18.9)	−3.6 (−7.3, 0.1)	.057
3 + medications	19.5 (16.0, 23.0)	16.2 (12.7, 19.8)	−3.2 (−5.4, −1.1)	.004*
Nausea severity				
0 medications	1.9 (0.8, 3.5)	1.6 (0.7, 3.1)	−0.3 (−1.4, 0.9)	.658
1–2 medications	1.8 (0.8, 3.3)	2.0 (0.9, 3.6)	0.2 (−1.0, 1.4)	.745
3 + medications	1.6 (1.1, 2.3)	0.7 (0.4, 1.2)	−0.9 (−1.4, −0.4)	.001*
Parent reported:				
QOL physical functioning				
0 medications	38.2 (15.0, 61.5)	50.1 (26.8, 73.3)	11.8 (−5.0, 28.6)	.162
1–2 medications	50.9 (27.6, 74.1)	37.0 (9.0, 65.1)	−13.8 (−36.9, 9.2)	.230
3 + medications	46.8 (35.6, 58.0)	60.6 (48.4, 72.7)	13.8 (3.6, 24.0)	.010*
QOL psychosocial				
Female	48.3 (41.3, 55.4)	56.8 (48.6, 64.9)	8.5 (0.3, 16.6)	.043*
Male	42.3 (23.6, 61.0)	71.8 (55.1, 88.6)	29.5 (11.5, 47.6)	.002*
Demonstrated for select ages				
Functional disability inventory				
12 years	23.8 (11.4, 49.7)	8.0 (3.7, 17.2)	−15.7 (−29.4, −2.1)	.025*
15 years	16.5 (11.4, 23.9)	10.8 (7.4, 15.8)	−5.7 (−9.9, −1.5)	.009*
18 years	11.5 (6.2, 21.4)	14.6 (7.7, 27.5)	3.1 (−3.1, 9.3)	.319
Child somatization inventory				
12 years	31.2 (14.2, 48.1)	9.9 (−7.6, 27.5)	−21.2 (−33.5, −8.9)	.001*
15 years	36.3 (27.8, 44.8)	24.2 (15.5, 32.8)	−12.1 (−18.2, −6.1)	<.001*
18 years	41.4 (27.1, 55.8)	38.4 (23.8, 53.0)	−3.0 (−13.3, 7.3)	.554

* *p* < .05.

^a^ *P*-value based significance on generalized mixed effects model (GLMM) with compound symmetry specified for repeat measures and variance components for random effect of patients. Normal distribution assumed for all outcomes with exception of FDI and Nausea which were positively skewed and gamma distribution with log link utilized (nausea scale included zero values, therefore, one was added to scale score and back-transformed for above presentation of estimates from model). Age treated as continuous variable in models.

Parent reported change in average FDI from baseline to week 4 non-significant after 15 years of age.

Parent reported change in average API from baseline to week 4 non-significant after 16 years of age.

Average improvement in QoL psychosocial score according to parent report was greater in male compared to female children (+29.5 points (95% CI 11.5, 47.6) vs. + 8.5 points (95% CI 0.3, 16.6), *p* < 0.05, respectively). On average, FDI and CSI scores decreased from baseline to Week 4 in an age-dependent manner with significance detectable in patients less than 15 and 16 years of age, respectively (Table 4).

Self-reported decrease in nausea severity across the 4-week period correlated with increased QoL physical functioning (*r*_s _= −0.558, *p* = 0.011). Decreased somatization (CSI) correlated with increased QoL psychosocial (*r*_s _= 0.457, *p* = 0.049). Decreased abdominal pain (API) correlated with decreased depression (*r*_s _= 0.381, *p* = 0.050). Parent observation of decreased API in their child also correlated with decreased child-reported depression (*r*_s _= .438, *p* = .037). Parent reported decrease in API, CSI, and FDI correlated with decreased child-reported anxiety (*r*_s _= 0.475, *r*_s _= 0.489, *r*_s _= 0.462, respectively, *p* < 0.05). Interestingly, decreased anxiety reported by child did not correlate with their self-reported change in API, CSI, and FDI (*p* > 0.05) (*r*_s_* = Spearman's correlation coefficient)* (Table 5).

**Table 5:** Correlation between change in abdominal pain (API), somatization (CSI), functional disability (FDI), and nausea severity (NSI) by child and parent report and child reported change in QOL and PROMIS outcomes.

*r*_s_	Δ in child-reported QOL				Δ in child-reported PROMIS		
	Physical functioning	Psychosocial	Generic core	Total	Anxiety	Depression	Global WB
Child-reported							
Δ API	.177	–.240	–.039	–.174	.314	.381∼	–.232
Δ CSI	–.392	–.457*	–.399	–.007	.127	.108	.063
Δ FDI	–.278	–.148	–.205	–.174	.212	.192	–.066
Δ NSI	–.558*	.229	–.436∼	.045	–.062	.231	–.306
Parent-reported							
Δ API	.054	.104	.174	–.225	.475*	.438*	–.052
Δ CSI	–.293	–.037	–.152	.131	.489*	.346	–.064
Δ FDI	–.125	.042	–.054	.108	.462*	.012	–.253
Δ NSI	–.251	–.180	–.262	–.172	.190	.348	–.137

Δ = Change (Week 4—Baseline).

*r*_s_ Spearman's correlation coefficient.

* *p* < .05, ∼*p* = .05.

Subset of patients with measurements in both time periods to assess within change for both metrics (*n* = 18 to 22).

When comparing the IBS group to the FD group for the child-report there was differences between the IBS and FD group (Table 6). Average nausea for the at baseline in IBS alone vs. FD alone was 1.4 (95% CI 0.9, 2.2) vs. 2.1 (95% CI 1.1, 3.6), respectively, *p* > .05. Decreased average nausea at four weeks was observed in both diagnosis groups and reached significance in the FD alone group (*p* < .05). Average physical functioning (QOL) at baseline in children with IBS alone appeared better than those with FD alone, although non-significant [52.4 (95% CI 39.0, 65.8) vs. 39.2 (95% CI 22.1, 56.3), *p* > .05]. Improvement at four weeks was observed in both groups (non-significant when examined within each group due to small sample size and relatively large variation). Average CSI was similar in diagnosis both groups at baseline: 43.8 (95% CI 33.0, 54.5) vs. 42.8 (95% CI 27.2, 58.3). Reduced average CSI at four weeks was significant and similar in both groups. The PROMIS anxiety from baseline to week 4 in the IBS group was 19.1 (95% CI 15.4, 22.7) to 15.7 (95% CI 12.0, 19.5), respectively, *p* > .05. For the FD group, the PROMIS global health child-report was reduced from 19.4 (95% CI 16.0, 22.7) to 21.3 (95% CI 18.0, 24.5), respectively, *p* > .05. The comparisons for the FD group vs. IBS in the parent-report also had notable differences between both groups (Table 7). The parent-report for the IBS group reached significance for change in QoL, FDI, CSI while the FD group did not.

**Table 6:** Examination of diagnosis (IBS alone vs. FD alone) effect on average change in quality of life (QOL), nausea, FDI, child somatization inventory, API, and PROMIS metrics from baseline to 4 weeks based on child responses, unadjusted.

	Child Dx IBS alone (*N* = 16)			Child Dx FD alone (*N* = 9)		
Outcomes	Baseline	Week 4	Difference	Baseline	Week 4	Difference
	Mean (95% CI)	Mean (95% CI)	(Wk4-base)	Mean (95% CI)	Mean (95% CI)	(Wk4-base)
			Mean (95% CI)			Mean (95% CI)
Child reported						
QOL						
Physical funct.	52.4 (39.0, 65.8)	50.4 (36.6, 64.2)	2.0 (−7.0, 11.1)	39.2 (22.1, 56.3)	45.7 (27.2, 64.1)	6.4 (−5.8, 18.7)
Psychosocial	54.5 (42.6, 66.4)	57.9 (45.5, 70.3)	3.4 (−6.6, 13.4)	54.8 (39.7, 69.9)	58.0 (41.2, 74.9)	3.2 (−10.3, 16.7)
G. Core scale	53.9 (42.4, 65.3)	55.1 (43.4, 66.9)	1.3 (−5.6, 8.2)	51.4 (36.7, 66.1)	53.1 (37.5, 68.7)	1.8 (−7.6, 11.1)
G. Well being	62.6 (53.0, 72.2)	57.5 (57.4, 67.7)	−5.1 (−4.5, 14.6)	60.9 (48.8, 73.1)	65.6 (51.7, 79.6)	4.7 (−8.2, 17.6)
Nausea severity	1.4 (0.9, 2.2)	1.1 (0.6, 1.8)	−0.3 (−0.8, 0.1)	2.1 (1.1, 3.6)	1.1 (0.4, 2.1)	−1.0 (−1.9, −0.2)*
FDI	16.0 (10.7, 24.0)	11.5 (7.5, 17.5)	−4.5 (−8.9, −0.2)*	21.0 (11.7, 37.8)	13.3 (7.5, 23.6)	−7.7 (−16.1, 0.7)
CSI	43.8 (33.0, 54.5)	25.1 (14.1, 36.1)	−18.6 (−25.2, −12.1)*	42.8 (27.2, 58.3)	25.1 (9.9, 40.3)	−17.6 (−26.9, −8.3)*
API	3.1 (2.5, 3.7)	1.8 (1.2, 2.4)	−1.2 (−1.8, −0.7)*	3.6 (2.7, 4.5)	2.0 (1.2, 2.9)	−1.6 (−2.4, −0.8)*
PROMIS						
Global health	19.9 (17.6, 22.2)	20.6 (18.2, 22.9)	0.7 (−0.6, 2.0)	19.4 (16.0, 22.7)	21.3 (18.0, 24.5)	1.9 (0.8, 3.7)*
Anxiety	19.1 (15.4, 22.7)	15.7 (12.0, 19.5)	−3.3 (−5.8, −0.8)*	18.0 (12.7, 23.3)	16.5 (11.4, 21.6)	−1.5 (−5.0, 2.1)
Depression	19.0 (14.7, 23.3)	15.1 (10.8, 19.5)	−3.9 (−7.9, 0.2)	16.1 (10.1, 22.2)	16.1 (10.1, 22.2)	0.0 (−5.6, 5.6)

* *p* < .05.

^a^ P-value based significance on generalized mixed effects model (GLMM) with compound symmetry specified for repeat measures and variance components for random effect of patients. Model included term for time point, diagnosis group, and an interaction effect between time point*diagnosis group. Normal distribution assumed for all outcomes with exception of FDI and Nausea which were positively skewed and gamma distribution with log link utilized (nausea scale included zero values, therefore, one was added to scale score and back-transformed for above presentation of estimates from model).

Pairwise contrast comparisons between diagnosis groups showed no significant difference within each time point for any outcome (*p* ≥ .05).

**Table 7:** Examination of diagnosis (IBS alone vs. FD alone) effect on average change in quality of life (QOL), nausea, FDI, child somatization inventory, API, and PROMIS metrics from baseline to 4 weeks based on parent responses, unadjusted.

	Child Dx IBS alone (*N* = 16)			Child Dx FD alone (*N* = 9)		
Outcomes	Baseline	Week 4	Difference	Baseline	Week 4	Difference
	Mean (95% CI)	Mean (95% CI)	(Wk4-base)	Mean (95% CI)	Mean (95% CI)	(Wk4-base)
			Mean (95% CI)			Mean (95% CI)
Child reported:						
QOL						
Physical funct.	47.7 (34.9, 60.5)	54.1 (40.1, 68.0)	6.4 (−9.0, 21.8)	41.8 (26.6, 57.0)	56.7 (38.2, 75.2)	14.9 (−4.2, 34.0)
Psychosocial	45.7 (36.0, 55.4)	60.4 (49.8, 70.9)	14.7 (3.0, 26.3)*	48.1 (37.2, 59.0)	54.7 (41.8, 67.6)	6.6 (−6.7, 19.8)
G. Core Scale	46.5 (36.4, 56.6)	58.1 (47.2, 69.0)	11.6 (0.2, 23.0)*	46.7 (34.6, 58.8)	55.4 (41.0, 70.0)	8.7 (−5.4, 22.8)
G. Well Being	52.1 (42.8, 61.3)	55.0 (45.3, 64.6)	2.9 (−7.6, 13.3)	53.8 (43.8, 61.7)	52.7 (40.1, 65.3)	−1.1 (−13.8, 11.5)
Nausea Severity	0.8 (0.3, 1.6)	0.6 (0.1, 1.4)	−0.2 (−0.9, 0.5)	1.4 (0.5, 2.9)	1.8 (0.8, 3.4)	0.4 (−0.8, 1.6)
FDI	18.3 (11.4, 29.2)	11.1 (6.8, 18.3)	−7.7 (−13.8, −0.4)*	14.5 (7.8, 27.1)	13.9 (7.5, 26.0)	−0.6 (−8.5, 7.3)
CSI	35.2 (21.8, 48.5)	20.1 (6.3, 33.9)	−15.0 (−24.0, −6.1)*	42.5 (25.5, 59.5)	33.1 (15.6, 50.5)	−9.4 (−20.6, 1.8)
API	3.1 (2.4, 3.9)	1.7 (0.9, 2.4)	−1.5 (−2.1, −0.8)*	3.4 (2.4, 4.3)	2.3 (1.3, 3.2)	−1.1 (−2.0, −0.2)*
PROMIS GH	19.4 (16.5, 22.2)	21.0 (18.1, 23.9)	1.6 (−0.1, 3.3)	17.8 (13.9, 21.8)	20.0 (16.1, 23.9)	2.2 (−0.1, 4.4)

* *p* < .05.

^a^ *P*-value based significance on generalized mixed effects model (GLMM) with compound symmetry specified for repeat measures and variance components for random effect of patients. Model included term for time point, diagnosis group, and an interaction effect between time point*diagnosis group. Normal distribution assumed for all outcomes with exception of FDI and Nausea which were positively skewed and gamma distribution with log link utilized (nausea scale included zero values, therefore, one was added to scale score and back-transformed for above presentation of estimates from model).

At baseline, both child-reported and parent-reported scores for the Generic Core Scale, Physical Functioning, and Psychosocial Health were significantly lower in the study cohort compared to healthy controls (Table 8). Following four weeks of PENFS treatment, a slight increase was observed in both child-reported and parent-reported scores across all categories. However, these scores remained significantly lower than those of healthy controls.

**Table 8:** Pedsql generic core scales scores of healthy controls compared to our study cohort at baseline and week 4, unadjusted.

	Healthy controls^a^	Study cohort	
		Baseline	Week 4
Child-reported	*N* = 936	*N* = 22	*N* = 22
Generic core scale	85.6 (11.9)	50.5 (18.3)*	52.3 (22.7)*
Physical functioning	89.6 (12.1)	43.9 (22.6)*	44.2 (22.4)*
Psychosocial health	83.5 (13.6)	52.8 (19.6)*	56.8 (24.9)*
Parent-reported	*N* = 1,106	*N* = 16	*N* = 16
Generic core scale	85.2 (12.8)	48.6 (15.8)*	59.7 (19.2)*
Physical functioning	88.7 (15.2)	48.4 (22.2)*	58.0 (17.7)*
Psychosocial health	83.3 (13.6)	49.0 (14.5)*	60.7 (20.4)*

^a^ Healthy controls data as reported by Varni JW et al. (3).

* *p* < .05 comparison of mean value in study cohort at each time point compared to healthy controls based on Welch's T-test of two independent samples with unequal variance.

## 4. Discussion

In this study, we present novel findings on the improvement of both symptomatology and QoL in pediatric patients with pain related DGBIs following PENFS treatment. Our investigation is distinct from previous research as it not only confirms the existing evidence supporting PENFS in alleviating symptoms such as pain, but also delves into the therapy's impact on QoL, particularly in the pediatric population (12).

The results of our study demonstrated significant associations between changes in symptom severity and QoL domains in pediatric patients with DGBIs undergoing PENFS therapy. A decrease in self-reported nausea severity over the four-week treatment period correlated with increased QoL physical functioning. This finding supports the notion that a reduction in nausea severity can lead to improvements in daily functioning, which may, in turn, contribute to enhanced QoL for patients with DGBIs. A recent study reported higher somatization scores, increased anxiety, and depression, and lower overall QoL in children with nausea, either with or without abdominal pain (14). Additionally, decreased somatization, as measured by the CSI, correlated with increased QoL psychosocial scores. This suggests that addressing somatization symptoms in pediatric patients with DGBIs may have a positive impact on their overall psychosocial well-being. In a study evaluating psychosocial distress measures in children with IBS, somatization was the strongest predictor for Psychosocial QoL (15). There is a significant role of somatization in predicting both abdominal pain frequency and persistence of pain in children with chronic abdominal pain (16, 17). Managing somatization is a complex process that involves both psychological and physiological factors. Therefore, a comprehensive approach that incorporates both non-pharmacological and pharmacological interventions is essential to effectively manage symptoms and enhance QoL. Despite improvements in GI symptoms, functioning, somatization and the QoL scores of DGBIs patients following PENFS therapy remained significantly lower than those of healthy controls. This highlights the enduring impact of DGBIs on children's QoL, even with treatment.

A unique aspect of our study is the detailed exploration of the parent-patient dyad relationship, which offers valuable insights into the perception of treatment outcomes from both perspectives. In our study, the parents reported better QoL scores than the children themselves. Prior studies have found imperfect agreement when the parental reporting of QoL was compared to that of children with chronic health conditions (18, 19). Rather than assuming the validity of one viewpoint, it is essential to take both perspectives into account and consider them equally (20). In clinical practice and trials, it is crucial to consider the perspectives of both children and parents regarding QoL disparities in patients with DGBIs vs. healthy individuals. The contrast between child self-reported and parent proxy-reported QoL underscores this necessity, which is in line with recommendations in the broader HRQOL literature (21). In our study, parent-reported decreases in API, CSI, and FDI correlated with decreased child-reported anxiety, no significant correlation was found between decreased anxiety reported by the child and their self-reported changes in API, CSI, and FDI. This discrepancy may be attributed to several factors, such as differences in the perception of symptom severity and improvement, or the influence of parental expectations on treatment outcomes or the presence of co-existing depression in patients. It may also suggest that anxiety, as a psychological symptom, might require a different approach to treatment, potentially involving cognitive-behavioral therapies or other psychological interventions.

Our study also revealed a significant correlation between decreased abdominal pain intensity (API) and decreased depression. Furthermore, parent observation of decreased API in their child correlated with decreased child-reported depression. Pain-predominant DGBIs have been shown to be more common in children with anxiety or depression compared with controls (22). Our findings highlight the potential bidirectional relationship between pain and depression in pediatric DGBIs patients, emphasizing the importance of addressing both physical and psychological symptoms to optimize treatment outcomes (7).

The reported QoL trajectories for children diagnosed with IBS and FD, seem to converge. This confluence suggests that, irrespective of their diagnostic labels, the subjective well-being of these children is comparably impacted. Nausea, a frequent accompaniment of DGBIs, manifested more prominently in children FD at baseline. This distinction, still present by week 4, delineates the necessity for tailored therapeutic interventions, especially when addressing nausea. Somatization, often linked with a heightened perception of bodily sensations, displayed interesting trends. Children with IBS reported more somatization at baseline compared to those with FD. Yet, when observing the shifts from baseline to week 4, both groups demonstrated statistically significant changes, potentially reflecting the variable nature of symptom progression in these conditions. Intriguingly, this pattern was somewhat reversed in the parent-reports, wherein initial somatization was less pronounced in the IBS group but displayed significant shifts by week 4. Functional disability, as assessed in our study, illuminates the unique experiences in children with IBS and FD. Children's self-reports favored a more profound disability experience in FD, while parent observations tilted towards IBS. Such dichotomies, inherent to DGBIs, accentuate the intricate interplay of perceptions, experiences, and reports between patients and caregivers. These differential responses across IBS and FD subgroups underscore the need for a more stratified approach in managing these conditions.

The observed associations between changes in symptom severity and QoL domains underscore the potential benefits of PENFS therapy for pediatric patients with DGBIs. Our findings emphasize the importance of a multi-modal treatment approach that addresses both physical and psychological symptoms to enhance QoL and overall well-being. Further research is needed to confirm these findings and to explore the optimal treatment strategies for pediatric DGBIs patients, considering the complex interplay between various symptoms and their impact on QoL.

Our findings align with recent literature examining the gut-brain axis and the role of the vagus nerve in pain modulation and inflammation. Current research has explored the use of vagal nerve stimulation in managing complex pain disorders, headaches, fibromyalgia, functional abdominal pain, constipation, and dyspepsia (10–12, 23, 24). Our study not only supports the positive effects of PENFS on pain reduction but also highlights its potential to improve patients' functionality in society, as evidenced by significant parent-reported improvements in QoL across multiple domains.

Moreover, our study emphasizes the need for a multi-modal treatment approach in patients with DGBIs. We observed that patients on three or more medications experienced significant reductions in anxiety and nausea severity, as well as improvements in QoL physical functioning according to parent reports. This may suggest that combining pharmacological and non-pharmacological interventions may be beneficial for some patients in managing their symptoms and enhancing their QoL.

## 5. Future directions and clinical implications

However, our study has several limitations, including a relatively small sample size and the absence of a sham control group. Future research should employ larger sample sizes, placebo-controlled study designs, and long-term follow-up data to evaluate the effectiveness of PENFS therapy compared to other treatment modalities and to assess the durability of treatment effects and optimal duration of therapy. These investigations can also explore the potential need for maintenance therapy or repeated courses of treatment or concurrent treatment with other modalities to ensure sustained improvements in symptomatology and QoL for pediatric patients with DGBIs. It is essential to examine the long-term effects of PENFS therapy on pediatric patients. Future studies should also investigate the role of patient-specific factors, such as duration of illness and co-morbidities, in determining treatment response and QoL outcomes.

Considering the observed discrepancies between parent and child-reported outcomes, future research should consider employing more objective measures of improvement, such as changes in grades, participation in extracurricular activities, or the number of missed events before and after PENFS therapy. Understanding the parent-child dyad and the factors that contribute to discrepancies in their perceptions of treatment outcomes could help inform the development of more effective communication strategies and tailored interventions that consider both the patient's and the parent's perspectives. This may further enhance our understanding of how both perspectives can be utilized in evaluating treatment progress and tailoring interventions to the specific needs of pediatric patients with DGBIs. Furthermore, there has been increasing interest in examining the microbiome of patients with various pain disorders, However, there is a lack of studies in the pediatric population. A recent study evaluated changes in the microbiome of adolescents with IBS after PENFS treatment, The microbiome showed decreased Clostridial species and long chain fatty acid (LCFA) microbial pathways post treatment (25). A future goal could be to examine the microbiome of children with other DBGIs besides IBS and changes in the microbiota secondary to PENFS.

In conclusion, the results of this study suggest that PENFS therapy is a promising non-pharmacological treatment for pediatric patients with DGBIs, potentially leading to improvements in both symptom severity and QoL. The observed correlations between changes in symptoms and QoL domains, as well as the interesting findings regarding the parent-child dyad, highlight the need for a comprehensive and multi-modal approach to the management of pediatric DGBIs patients. Further research is needed to confirm these findings, elucidate the mechanisms underlying the observed associations, our understanding of the gut-brain axis and optimize treatment strategies, to provide personalized and effective care for pediatric patients with DGBIs, ultimately improving their QoL and ability to thrive in society.

## Data Availability

The raw data supporting the conclusions of this article will be made available by the authors, without undue reservation.
