# Supplementary material for: Prospective study of the effect of auricular percutaneous electrical nerve field stimulation on quality of life in children with pain related disorders of gut-brain interaction
Source: Front Pain Res (Lausanne). 2023 Sep 8;4:1223932. doi: 10.3389/fpain.2023.1223932 (PMC10515210; doi:10.3389/fpain.2023.1223932)
Supplement: Supplementary file 1 [file Datasheet1.pdf]

PENFS and QoL

Prospective Study of the Effect of Auricular Percutaneous Electrical Nerve Field Stimulation on Quality of Life in Children with Pain Related Disorders of Gut-Brain Interaction

1 HOW MANY CHILDREN SUFFER FROM DGBIs

Disorders of the Gut-Brain Interaction (DGBIs) account for 50% of pediatric gastrointestinal (GI) consultations.

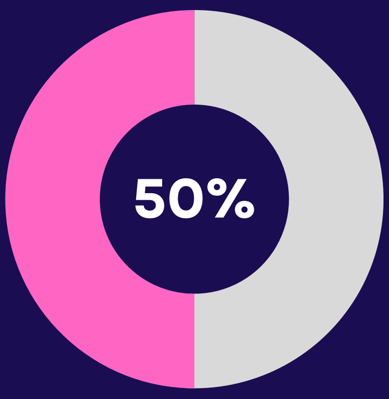

2 HOW DO DGBIS AFFECT THE QOL AND MENTAL HEALTH IN CHILDREN

Pediatric DGBIs patients, especially those with chronic abdominal pain (AP), have impaired (QoL) quality of life and increased psychological distress in the form of anxiety and depression.

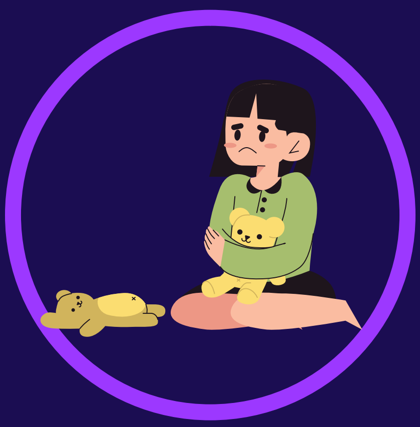

3 WHAT IS THE PRIOR EVIDENCE THAT PENFS HAS WORKED FOR DGBIs

Percutaneous Electrical Nerve Field Stimulation (PENFS) therapy has been shown to be effective in improving symptoms and functioning in children with DGBIs.

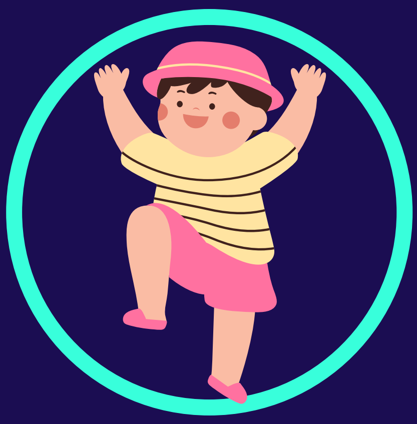

4 WHAT WERE THE METHODS FOR THIS STUDY

Application of PENFS was done on children with DGBIs and between the ages of 11-18 years old. Auricular PENFS therapy was done once a week for 4 consecutive weeks alongside questionnaires.

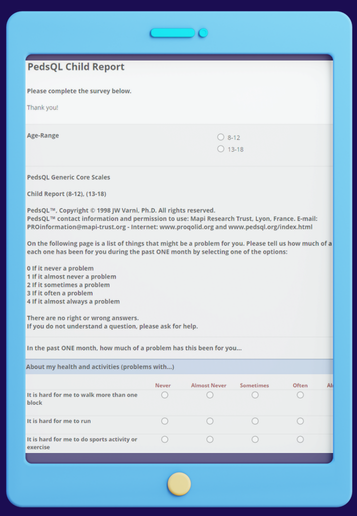

5 WHAT WERE THE RESULTS OF THIS STUDY

- This study included 31 patients with an average age of 15.7 years old.
- Of the 31 patients, 80.6% were female.
- After PENFS therapy, patients reported significant reductions in abdominal pain, nausea severity, functional disability, somatization, and anxiety from baseline to week 4.
- Parents reported significant improvement in their child's quality of life regarding physical function, psychosocial function, and generic core scale scores.
- Average scores on the Patient-Reported Outcomes Measurement Information System (PROMIS) Global Health scale significantly improved based on both patient and parent report.

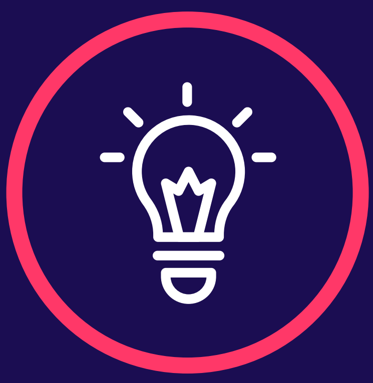

Ashish Chogle, MD, MPH[1], Kaajal Visnagra, MD[2], Jamie Janchoi, BS[1,3], Tammy Tran, MSN[1], Rachel Davis, PhD[3], Nicole Callas, BSN[1], Elisa Ornelas, BA [1,3]

Infographic: Prospective Study of the Effect of Auricular Percutaneous Electrical Nerve Field Stimulation on Quality of Life in Children with Pain Related Disorders of Gut-Brain Interaction

Copyright © 2023 CHOC Children's
